# Making X-ray crystallographic refinement accessible for non structural biologists

**DOI:** 10.1063/4.0000972

**Published:** 2025-10-27

**Authors:** Miki Senda, Toshiya Senda

**Affiliations:** KEK, Tsukuba, Ibaraki, Japan

## Abstract

In X-ray crystallographic structure analysis, crystallographic refinement is often performed by experts, and the resulting coordinates are used as- is by collaborators. However, to obtain a highly reliable model and its interpretations, it is essential that not only experts in crystallographic structure analysis but also members involved in the project actively participate in model building and evaluation of the final coordinates. Here, we present our efforts to enable non structural biologists to check electron density maps and perform crystallographic refinement with the support of X-ray crystallographers.

First, we would like to introduce two examples where this attempt was successful. The first example is a graduate student from a collaborating laboratory who became capable of crystallographic refinement by continuing weekly one-hour Zoom meetings (starting with tutorials and then working on their own project) for 1-2 months. The second example is an undergraduate student who came for an internship and, by staying in the laboratory for about three months and starting with tutorials, was able to perform crystallographic refinement for a project being conducted in collaboration and register two structures in the PDB. Through these attempts, we have developed tutorials consisting of three parts: "Let's use Coot," "Let's perform crystallographic refinement," and "Let's evaluate the structure using the PDB validation tool."

To start, we created a tutorial called "Let's use Coot" to allow users to display and view the electron density map along with the model. This tutorial begins with a brief explanation of the X-ray crystallographic structure analysis workflow, followed by an understanding of the contents of .pdb and .mtz files. Then, using the complex of shigatoxin (STX) and an inhibitory peptide (REF, PDB ID = 7D6R), which our group has refined and already published, as an example, users are guided through the process of displaying the model and map. Additionally, we provide brief explanations of frequently used functions and types of electron density maps. This enables users to display the model and electron density map using Coot and focus on areas of interest to check the structure. Next, users are guided through the process of checking the model and electron density map of their target protein. Although it is simple, as it only requires applying the procedure learned with STX to their research target protein, this step allows them to understand that the clarity of the electron density map varies depending on the protein's location and that the model's reliability differs by location.

In the second tutorial, "Let's perform crystallographic refinement," we propose a directory structure in their computer and note-taking methods, and then guide users through the process of copying tutorial data to the appropriate directory and performing crystallographic refinement of the STX and inhibitory peptide complex (REF, PDB ID = 7D6R).

The third tutorial, "Let's evaluate the structure using the PDB validation tool," shows the procedure for checking the reliability of the structure refined by the user and modifying the structure as necessary.

We believe that these tutorials will contribute to making X-ray crystallography more accessible and accelerating structural biology research.
